# Accessing complex reconstructed material structures with hybrid global optimization accelerated *via* on-the-fly machine learning†

**DOI:** 10.1039/d3sc02974c

**Published:** 2023-07-20

**Authors:** Xiangcheng Shi, Dongfang Cheng, Ran Zhao, Gong Zhang, Shican Wu, Shiyu Zhen, Zhi-Jian Zhao, Jinlong Gong

**Affiliations:** a School of Chemical Engineering and Technology, Key Laboratory for Green Chemical Technology of Ministry of Education, Tianjin University Tianjin 300072 China zjzhao@tju.edu.cn jlgong@tju.edu.cn; b Collaborative Innovation Center of Chemical Science and Engineering (Tianjin) Tianjin 300072 China; c Haihe Laboratory of Sustainable Chemical Transformations Tianjin 300192 China; d National Industry-Education Platform of Energy Storage, Tianjin University 135 Yaguan Road Tianjin 300350 China; e Joint School of National University of Singapore and Tianjin University, International Campus of Tianjin University Binhai New City Fuzhou 350207 Fujian China; f Department of Chemistry, National University of Singapore 3 Science Drive 3 Singapore 117543 Republic of Singapore

## Abstract

The complex reconstructed structure of materials can be revealed by global optimization. This paper describes a hybrid evolutionary algorithm (HEA) that combines differential evolution and genetic algorithms with a multi-tribe framework. An on-the-fly machine learning calculator is adopted to expedite the identification of low-lying structures. With a superior performance to other well-established methods, we further demonstrate its efficacy by optimizing the complex oxidized surface of Pt/Pd/Cu with different facets under (4 × 4) periodicity. The obtained structures are consistent with experimental results and are energetically lower than the previously presented model.

## Abstract 2

This paper describes a multi-tribe hybrid evolutionary algorithm that combines differential evolution and genetic algorithms for surface structural optimization accelerated by an on-the-fly machine learning calculator.

## Graphical abstract

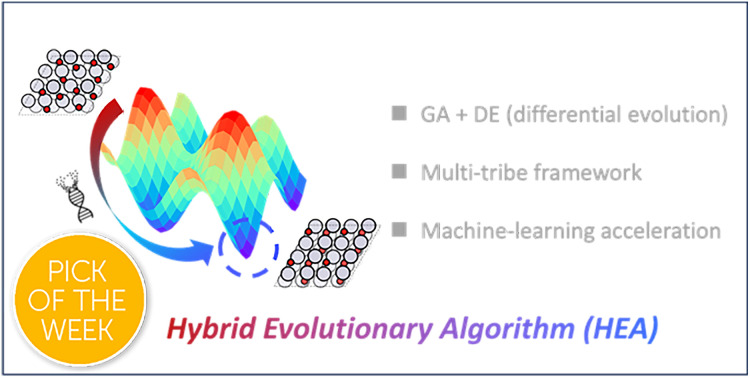

## Introduction

The rational design of highly efficient materials requires an atom-level understanding of their structure–performance relationship.^1–5^ However, under working conditions, most materials undergo a structural reconstruction accompanied by an unpredictable performance.^6–9^ For example, some bimetallic catalysts like Au–Ag alloys can exhibit dynamic geometrical and compositional reconstruction during the reaction, which generates active sites to boost performance.^6,10–14^ In contrast, under high voltage, metal catalysts can be partially oxidized, which results in destabilization and higher dissolution of the active species.^15,16^ Surface reconstruction sensitively varies with the nature of unreconstructed surfaces and the compositions/concentrations of adsorbent.^17,18^ Due to the difficulty to model a reconstructed surface, it is a generally adopted, but improper, practice to oversimplify a complex surface when modeling. For example, to model a partially oxidized surface, some studies place adsorbed oxygen,^19^ or only a layer of metal oxides,^20^ upon a metal surface without considering reconstruction.^21^ To achieve the rational design of highly efficient materials, it is essential to properly model their reconstructed structures to understand their real structure–performance relationship.

Reconstructed surfaces tend to adopt the most thermodynamically stable structures,^22^ which is the lowest point on the potential energy surface (PES), the so-called global minimum (GM).^18^ Finding the GM of a working material constitutes a global optimization (GO) problem. There has been significant progress in developing GO algorithms for chemical structure optimization. However, most of them are mainly developed for isolated particles like crystals, clusters, or supported clusters, with simplified models deemed to be sufficient for most investigations.^22–24^ The optimization of the surface system is more geometrically restricted than that of isolated particles, owing to the periodicity and the presence of strong covalent bonds between surface atoms and the underlying support,^25^ and generally more atoms are required for reliable modeling.

Two major difficulties should be considered for globally optimizing surface structures: how to efficiently explore the highly complex PES, and how to reduce high computational costs caused by massive local relaxations used to describe the PES.^26^ Indeed, some GO algorithms that were originally developed for isolated particles have been applied to surface structures.^27,28^ However, their efficiency has not yet been examined systematically. Previous reports have expressed concern about the efficiency of the genetic algorithm (GA) that two good parent structures may produce poor candidates with high energy.^29,30^ The insufficient efficiency also limits the model size for optimizing a surface system, as most studies are conducted generally with no more than (2 × 2) periodicity.^31–37^ It is even unreliable since no known criterion guarantees that the “best structure” encountered by a GO algorithm is the GM that reflects the reconstructed structures.^38^

The absence of a criterion is primarily attributed to the inherent limitations in the spatiotemporal resolution of characterization techniques, resulting in a dearth of prior knowledge regarding the precise atomic-level structure of a reconstructed surface.^39^ This situation poses a significant challenge to the integration of machine learning (ML) aimed at mitigating the computational burden associated with GO. It is dangerous to rely solely on a pre-trained ML calculator that suffers extrapolation problems, as the GM can correspond to a very narrow basin of the PES and can hardly be involved in the training set.^40,41^ Previous research on ML-involved structural searches, using techniques without extrapolation design, such as neural networks, has generally been randomly generated or an already built database,^42^ or adjusting the atomic position is very constrained during the search.^43–45^ Even for on-the-fly ML frameworks with sampling design such as Bayesian optimization,^46^ if they are applied solely without incorporating near-GM structural features into the training set, their effectiveness can be compromised.^47^

This paper describes a new strategy for the global optimization of complex catalytic surfaces using a hybrid evolutionary algorithm (HEA) that combines differential evolution (DE) and genetic algorithms with a co-evolution framework. An on-the-fly machine learning calculator based on Gaussian processes is adopted to complement local evaluations and expedite the identification of low-lying structures. We demonstrate the HEA method in obtaining the complex surface oxide structure of different facets of transition metals like Pt, Pd, and Cu using a (4 × 4) supercell. The globally optimized structures are lower than previously reported theoretical modeling and are consistent with experimental observation, providing important clues for the rational design of catalysts.

## HEA methods

**Fig. 1:**
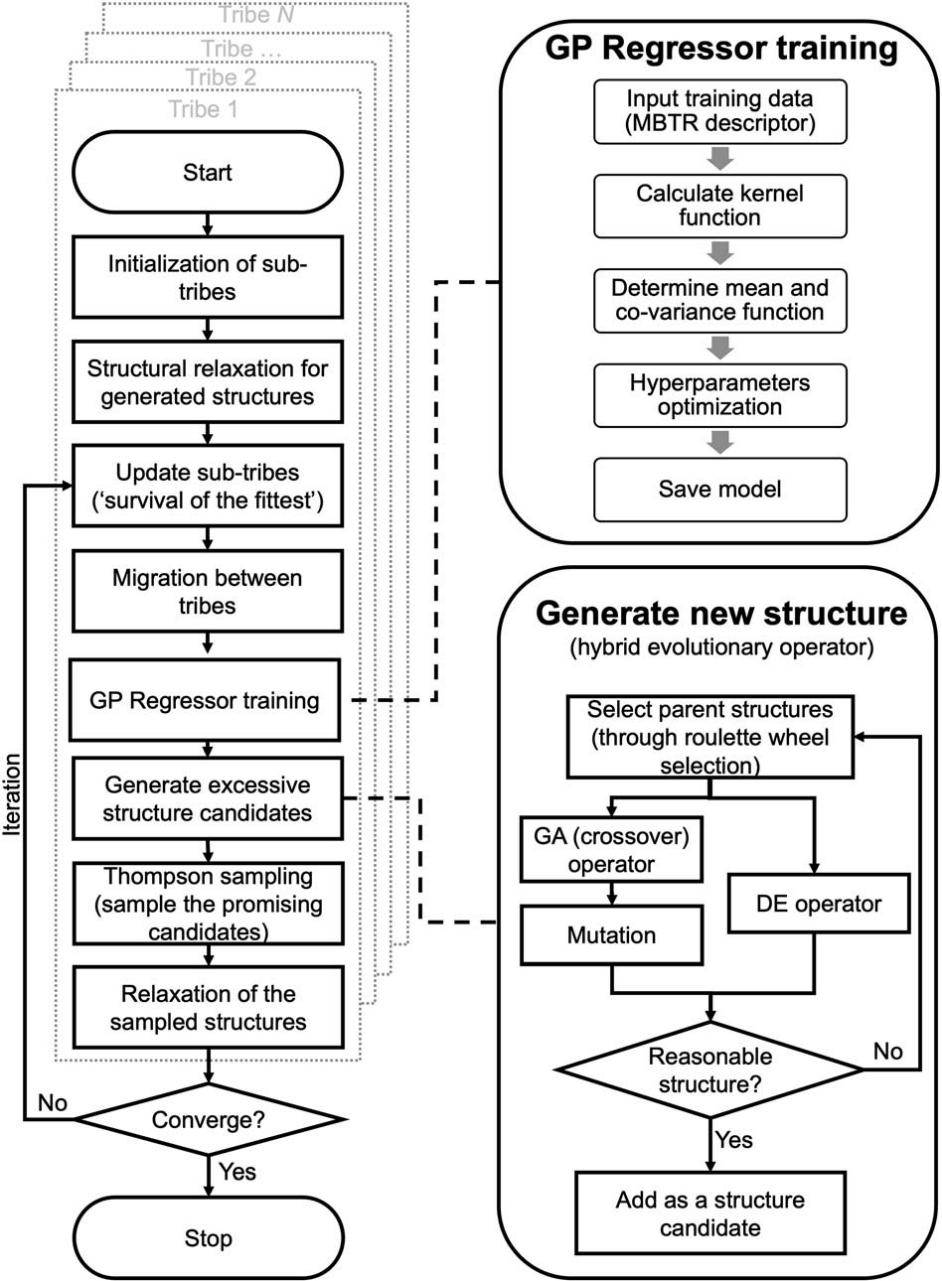
Overview of the workflow in the HEA program.

A flowchart of the HEA program is shown in Fig. 1. A “tribe” framework is adopted in the HEA program to simulate the real evolutionary process in nature. Specifically, several optimization processes (each is considered as a “tribe”) are concurrently run with a periodic exchange of the most stable members among tribes. Firstly, an initial set of structures is generated at random within appropriate limits for a (small) cell shape and interatomic distances, which is then enlarged to the required periodicity and relaxed at the DFT level. Thus, the generated structure has high symmetry and is more likely to be stable.^48,49^ An ML calculator based on a Gaussian process (GP) regressor is trained on-the-fly using relaxed structures to expedite the identification of low-lying structures, separately for each tribe.

For the offspring population in each tribe, excessive offspring candidates will be generated by a hybrid evolutionary operator: one is to produce offspring candidates from two-parent structures by the GA operator introduced by Deaven and Ho.^29^ Note that mutations (permutation, rattle, and mirror) are applied with pre-set probability for a newly generated structure.^51^ Another is to perform the DE operator, and three strategies are introduced:^52^

1 *X*_de_ = *X*_*r*_3__ + *F* × (*X*_*r*_1__ − *X*_*r*_2__)

2 *X*_de_ = *X*_best_ + *F* × (*X*_*r*_1__ − *X*_*r*_2__)

3 *X*_de_ = *X*_*r*_3__ + *F* × (*X*_best_ − *X*_*r*_3__) + *F* × (*X*_*r*_1__ − *X*_*r*_2__)

where the new structure *X*_de_ is generated by a linear combination between several randomly selected parent structures (*X*_*r*_1__, *X*_*r*_2__, *X*_*r*_3__,…) and a scaled difference (controlled by scaling factor *F* ∈ [0, 2]) between other donor structures. *X*_best_ represents the most stable structures in the parent population. Eqn (1)–(3) can be denoted as “DE/rand/1”, “DE/best/1” and “DE/rand-to-best/1” respectively.

The generated candidates will first be evaluated by the GP regressor. In practice, the GP regressor needs to deal with an unrelaxed structure. Considering time consumption and memory problems for force prediction, the GP regressor is directly trained and performed using unrelaxed structures with their relaxed energy.^47,53,54^

The GP regressor was implemented using the GPyTorch Python library.^55^ A Gaussian process uses Bayesian inference and assumes that the prior distribution for the data can be given by a multivariate normal distribution, while its task is to infer the Gaussian posterior distribution *p*(E_*_|*X*,*y*,X_*_) for the unseen datapoint X_*_ (waiting for exploration) based on the observed training set (*X*, *E*_*θ*_). *X* is taken to be the feature vector rather than the Cartesian coordinates, where the many-body tensor representation (MBTR) descriptor is adopted by the DSCRIBE software package.^50,56^ MBTR descriptors provide whole-system representations of periodic systems with the locality of chemical interactions being exploited. To reduce complexity for representing large surface systems, only the top three layers (that are more prominent to reconstruct) are selected for training, while the bottom layer (representing the bulk structure) is excluded. For more realistic modeling, the target value *y* differs from *E*_*θ*_ by adding an independent identically distributed Gaussian noise, 
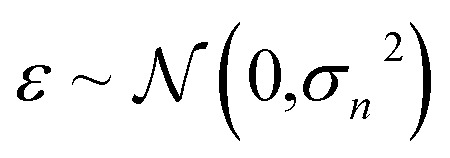
. The key predictive equations for the GP regressor are^57–59^4
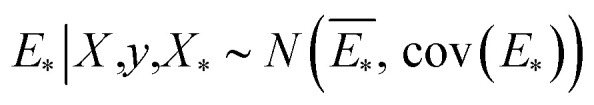
where5
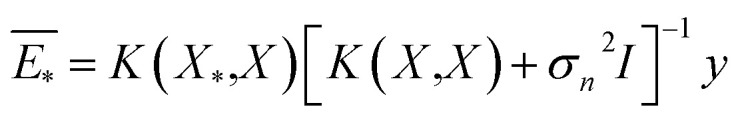

6 cov(E_*_) = *K*(X_*_,*X*) − *K*(X_*_,*X*)[*K*(*X*,*X*) + *σ*_*n*_^2^*I*]^−1^*K*(X_*_,*X*)

The predictive mean 
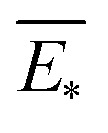
 of the distribution is the estimation of the potential energy, while the variance cov(E_*_) can be an uncertainty quantification. *K*(·,·) denotes the covariance function (or kernel) matrix that is used to characterize the similarity between samples, which is the very heart of the GP regressor. In our study, the covariance function was chosen to be a sum of a Matern kernel and a linear kernel as7
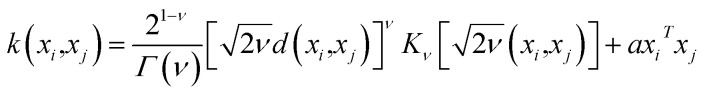
where *Γ* is the gamma function, *d*(*x*_*i*_,*x*_*j*_) is the scaled distance between *x*_*i*_ and *x*_*j*_, *ν* is a smoothness parameter where smaller values are less smooth, *K*_*ν*_ is a modified Bessel function and a variance parameter. The Matern kernel is a generalization of the Gaussian kernel that has proved to have an advantage in high dimensional inputs,^60,61^ and its synergy effects with the MBTR descriptor have been proven previously.^50^

The optimal hyperparameters Θ* are determined by the log marginal likelihood as

8 Θ* = arg max log *p*(*y*|*X*,*Θ*)

The Thompson sampling (TS) method is used as the acquisition function that leverages the uncertainty in the posterior to guide exploration, a randomized strategy that samples a reward function (that is relative to potential energy) from the posterior and queries the structure *x*_*n*+1_ with the highest reward.^62,63^ Fig. S1† shows an illustrative example of a TS-guided on-the-fly structure search in a non-convex search space.

9 *x*_*n*+1_ = arg max *f*_*w*_(*a*) where *w* ∼ *p*(*y*|*X*,*Θ*)

Equipped with this trained GP regressor, we choose a batch of multiple candidates, namely the batch Thompson sampling (B-TS) method. Different from the lower confidence bound (UCB) function used in similar studies,^47,59,64^ the B-TS method can naturally trade-off between the exploration and exploitation of the PES with no free parameters, thus avoiding the damage of efficiency caused by an inappropriate parameters setting of the UCB function,^41^ the effectiveness of which in searching chemical space has been demonstrated and reported before.^63^

Only these “most promising” structures are evaluated at the DFT level. The population is then updated under the ‘survival of the fittest’: a certain number of the most stable structures from the current (parent + offspring) population are kept, while others are eliminated.^22^ Nonetheless, all DFT-evaluated structures are added to the training dataset, and the GP regressor is re-trained on-the-fly.

## Results and discussion

### Performance of the HEA method

**Fig. 2:**
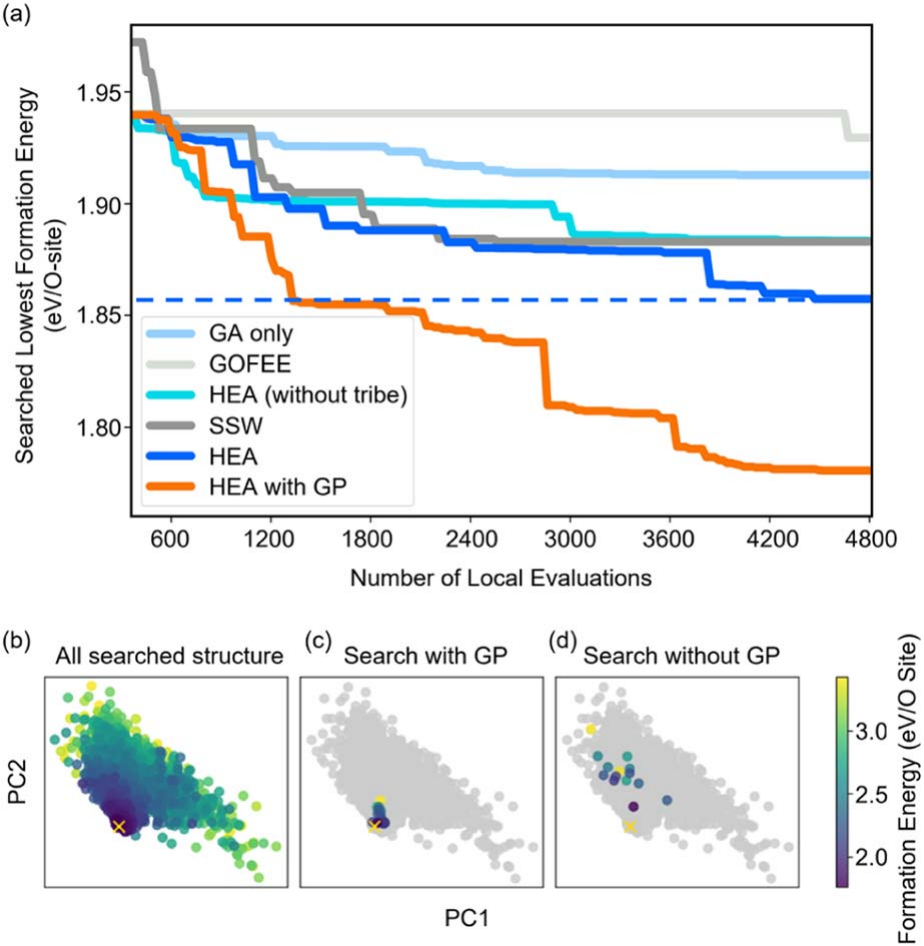
(a) Structural optimization performance of (4 × 4) 0.75 ML O–Pt(111) among HEA and other well-established methods. All results are repeated three times, and the plotted line represents the mean value. (b) Principal component analysis (PCA) visualization of the MBTR descriptor of the searched structures along the first two PCs (preserving 87% of the dataset variance) visited in the 10th generation in independent searches with/without the GP regressor. The yellow ‘x’ represents the GM the HEA program finally found.

Fig. 2(a) shows the optimizing performance of (4 × 4) 0.75 ML O–Pt(111) as a function of the number of local evaluations, among HEA (with different settings) and other well-established methods, such as GOFEE^59^ and SSW,^65^ for surface systems, where the HEA program (with and without the GP regressor) achieves the highest performance. With the on-the-fly GP regressor, the HEA program can be further accelerated: the number of local evaluations decreased almost threefold (which saves around 3600 local evaluations) to reach the same energy level, and the GM found eventually is much lower. A dimensionally-reduced visualization of this accelerated performance is presented in Fig. 2(b), showing that the search with the GP regressor is closer to the GM region than that without the GP regressor after a certain number of generations. The superior performance of the HEA program is also presented in Fig. S3(a)† for the optimization of the (2 × 2) surface. After the supercell, the obtained (2 × 2) structure is 0.28 eV per O-site less stable than the optimized (4 × 4) structure, shown by the green dot of Fig. 4(a), highlighting the necessity of directly optimizing in a bigger periodicity. As the structural search for a (4 × 4) surface requires even 10 times more local relaxation than that of a (2 × 2) surface, an accelerated module like the GP regressor is desired.

**Fig. 3:**
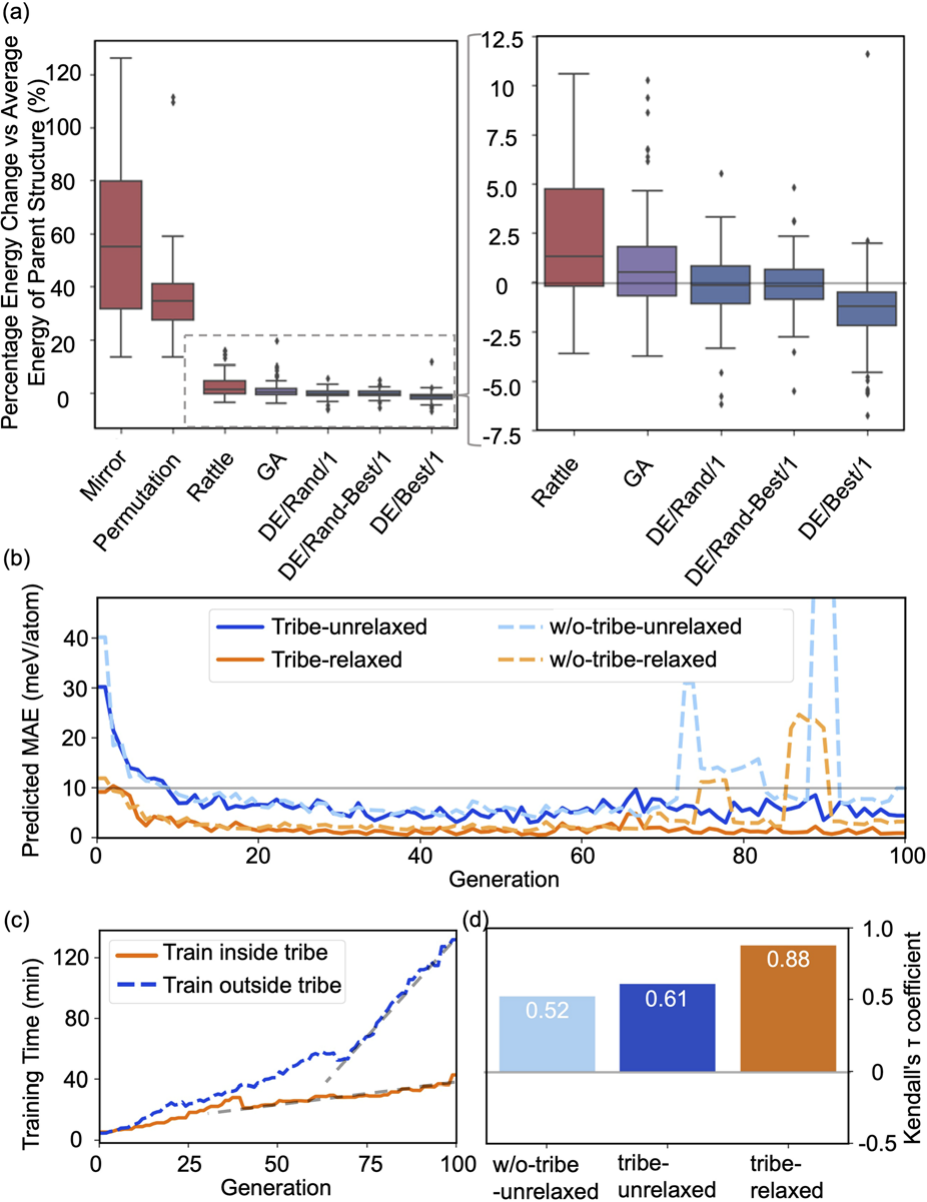
(a) Boxplots of energy changes compared to the average energy of the parent structures for different offspring operators in the HEA program. Details of the equation are provided in the ESI.† The lines inside the boxplots show the median energy change, the boxplots extend from the lower to the upper quartile of the data, whiskers extend from the 5th to the 95th percentile of the data, and black dots show data points outside of the whisker range. (b) The on-the-fly predict accuracy in optimizing O–Pt(111). “Tribe-unrelaxed”: train the GP regressor using unrelaxed structures with their relaxed energy separately inside each tribe, “-relaxed”: using relaxed structures, “w/o-tribe”: train only one GP regressor outside using structures from all tribes. The number of data points for the training set for 0, 20, 40, 60, and 80 generations is 360, 1260, 2160, 3060, and 3960, respectively, of which one-third are for “tribe” as three tribes are adopted. (c) Time spent for training the GP regressor inside or outside the tribe. (d) Kendall's *τ* coefficient of the ranking of all predicted energy and its relaxed energy collected from a HEA search. Details of Kendall's *τ* coefficient are provided in the ESI.†

The high efficiency of the HEA program stems from three features: firstly, in Fig. 3a and b, the newly introduced DE operators are much more effective and stable at generating lower energy configurations compared with the GA operator. Mirror and permutation mutation, which is widely used in the optimization of isolated particles, performs poorly when dealing with the surface system. As a result, in Fig. 2(a), GA only cannot efficiently optimize the (4 × 4) surface, which is exceeded by the HEA program combining both GA and DE operators. Secondly, the (on-the-fly) accuracy of the GP regressor produces reliable prediction of (unrelaxed) candidates. In Fig. 3(b), despite the accuracy loss, the MAE for both the models trained using relaxed and unrelaxed structures is below 10 meV per atom after around 10 generations (where the number of data points for the training set is 750).

A relatively correct ranking of the offspring candidates is achieved, as shown in Fig. 3(d) through Kendall's *τ* coefficient, demonstrating a reliable sampling during the search. Fig. 2(b) also reflects that the MBTR descriptor contains the relevant structural information for approximating the energy, which is the prerequisite for an accurate GP regressor. Finally, introducing the “tribe” framework not only helps maintain a high structural diversity for each tribe,^48^ but also enhances the efficiency of the GP regressor. Fig. 3(b and d) show that the accuracy is improved, and the sampling is more targeted. While the sparse GP regressor does not provide enough accuracy (Fig. S5†), here a GP regressor with a full covariance matrix is used that requires *o*(*N*^3^) computational time and *o*(*N*^2^) memory space for Cholesky decomposition. Thus, it becomes expensive and its numerical stability is degraded for a large dataset.^66,67^ Dividing the training dataset using the “tribe” framework naturally avoids these problems as shown in Fig. 3(c). All the above features contribute to the enhanced structural searchability of the HEA program.

### Application in reconstructed oxide structures

The efficacy of the HEA program is further demonstrated in modeling the reconstruction of metal oxides, knowledge of which has been limited because of their complexity and the scarcity of surface-sensitive characterization techniques.^18,68^ Global structural optimization is thus necessary to be applied. As a proof of concept, the complex surface oxides of different metals (Pt, Pd, and Cu) are studied here using the HEA program.

Pt can undergo irreversible restructuring under reaction conditions, due to the surface oxidation and subsequent Pt dissolution, which is thought to decrease catalytic efficiency and durability.^19,69–72^ However, the atomic-level modeling of the Pt oxidation remains uncertain.^73^

**Fig. 4:**
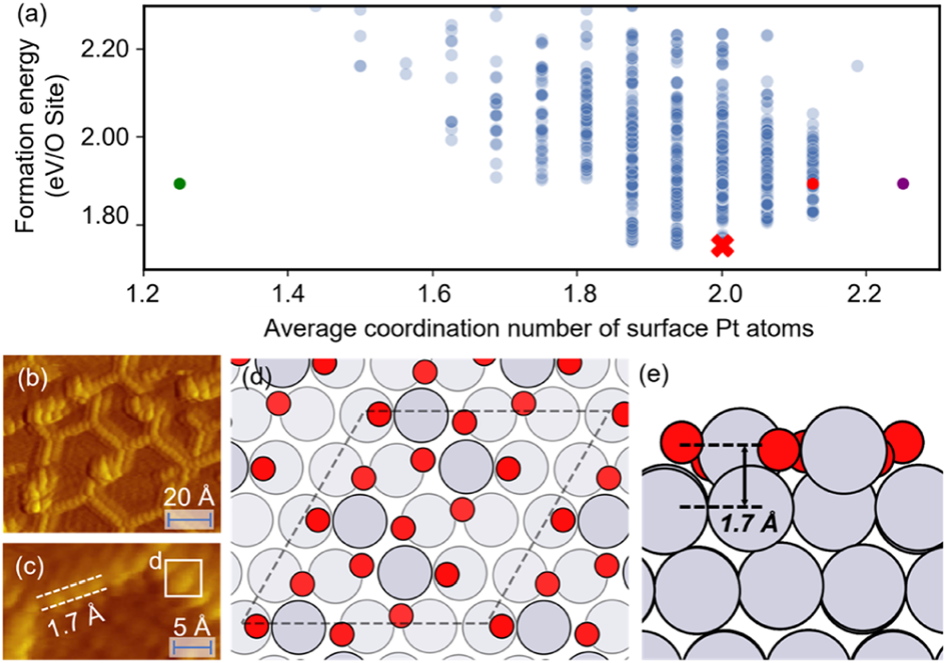
(a) The scatterplot of the energy of the structure search during a HEA search and the average coordination number of surface Pt atoms. Red cross: the globally optimized structure. Purple dot: the structure proposed by Hawkins *et al.*^74^ Red dot: the structure searched by LASP. Green dot: the (2 × 2) optimized structure (after supercell). (b and c) STM images of oxidized Pt(111) (reproduced with permission from ref. 75, Copyright 2008 Elsevier). (d and e) The globally optimized structures of (4 × 4) 0.75 ML O–Pt(111) through the HEA program.

The optimized structures of 0.75 ML O–Pt(111), which are thought to exist at around 1.0–1.2 *V*_RHE_ during electro-oxidation,^73,76^ are shown in Fig. 4(d and e). The structures consist of two interconnected, protruding square planar PtO_4_ units that are 1.7 Å in height. It is consistent with the scanning tunneling microscopy (STM) in Fig. 4(b and c) that the oxidized Pt(111) surface consists of a network of mono-atom-high (1.7 Å), worm-shaped islands.^75,77^ The surface oxidation state of PtO_4_ units is between that of PtO (Pt^2+^) and Pt_3_O_4_ (Pt^2.7+^), which exactly fits the *in situ* XANES showing that the oxidized Pt surface formed at >1.0 V presents square-planar PtO_4_ units with Pt in a slightly higher oxidation state than in PtO.^78^ With a similar oxidation state, the PtO_4_ units also resemble the PtO and Pt_3_O_4_ bulk oxide shown in Fig. S6.†

In Fig. 4(a), compared with the previously proposed O–Pt(111) model by Hawkins *et al.*,^74^ our optimized structure is 0.14 eV per O-site lower. Although Hawkins *et al.*'s model (Fig. S3†) also contained PtO_4_ units, the chain structures that are linked with each PtO_4_ unit are not as stable as the separated PtO_4_–PtO_4_ structures we obtained. Note there is no known criterion guaranteeing that the “best structure” encountered by a GO algorithm is the “true” global optimum.^38^ Nevertheless, a lower energy allows our obtained structure to have a greater possibility to be the most abundant and representative phase under reaction conditions.^22^

**Fig. 5:**
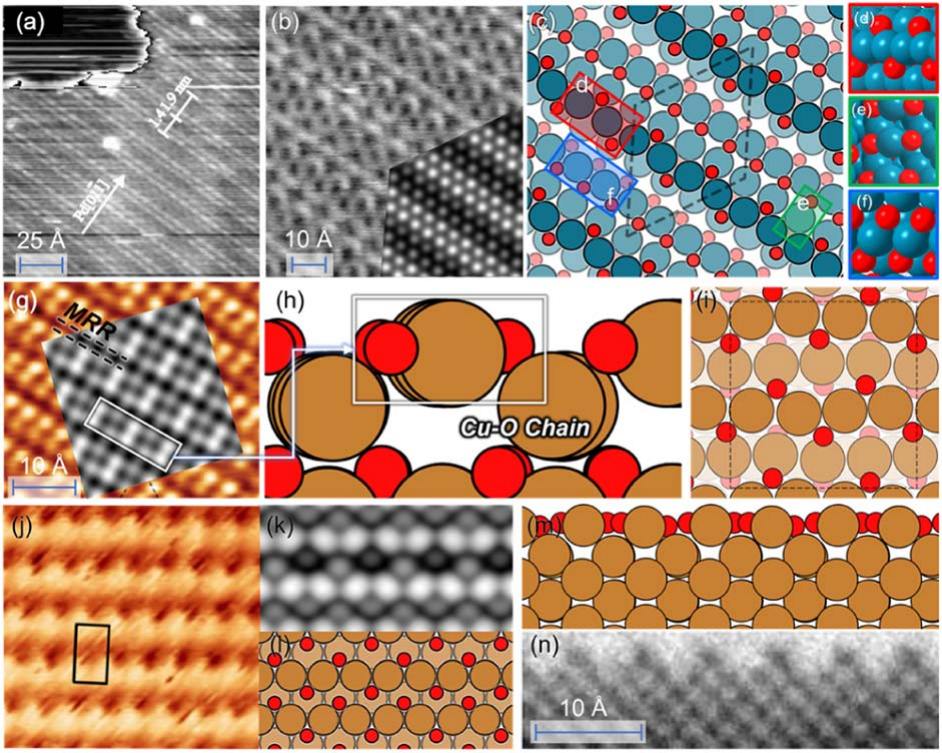
(a and b) STM image of oxidized Pd(111). (Reproduced with permission from ref. 79, Copyright 2000 IOP Publishing.) Inset: simulated STM images based on the optimized structure. (c–f) Optimized structures of 1 ML O–Pd(111). (g) STM images of O–Cu(100). (Reproduced with permission from ref. 80, Copyright 2013 American Chemical Society.) Inset: simulated STM images based on the optimized structure; (h and i) optimized structures of 1 ML O–Cu(100); (j) experimental STM images of Cu(110). (Reproduced with permission from ref. 81, Copyright 2014 Elsevier.); (k) the simulated STM images based on the globally optimized 1 ML O–Cu(110). (i–m) The obtained globally optimized surface structures of 1 ML O–Cu(110) through the HEA approach. (n) HRTEM image of reconstructed Cu(110) layers under O_2_ pressure (reproduced with permission from ref. 82, Copyright 2022 American Chemical Society).

Pd and Cu are widely used catalysts but both suffer from severe reconstruction under the reaction conditions.^83–85^ A typical characteristic for oxidized Pd(111) is the “Persian-carpet” pattern observed in STM, with which the simulated STM image based on the optimized structure is well consistent, as shown in Fig. 5b. Fig. 5c–f show that the optimized structure consists of several parallel chains with different features from the PdO_2_ to the PdO_4_ unit. Such multiple co-existing oxygen species have been observed by *in situ* XPS studies.^86^ Our optimized structure is 0.35 eV per O-site lower than the structure searched by USPEX as shown in Fig. S4,† and 0.61 eV per O-site lower than CALYPSO as reported previously.^87^ Compared with the CALYPSO model, we both presented the existence of subsurface O atoms that have been experimentally reported,^88^ while the CALYPSO model failed to further contain parallel chains that form a “Persian-carpet” pattern.

Fig. 5(h and i) show that the oxidized Cu(100) forms *via* the creation of the Cu–O chain that resembles the bulk Cu_2_O.^89^ Experimentally, a Cu_2_O signal has been observed during the initial oxide growth of Cu(100)^90^ along with the missing-row reconstruction (MRR) in Fig. 5(g),^82,91,92^ which is consistent with the simulated STM image based on our optimized structure. Such MRR is reported widely for Cu oxidation, which is caused by the increasing surface stress and has been previously proven to be energetically favorable.^82,91^ Subsurface oxygen is also contained in our structure, linking with the Cu–O chain through O–Cu–O units, which is believed to form above 0.5 ML O coverage experimentally,^92,93^ and to contribute to the increased stability of the MRR structure.^94^ Our optimized structure is 0.31 eV per atom more stable than the well-known (2√2 × √2)*R*45° reconstructed Cu(100) model^80,95^ that also presents an MRR structure but fails to reflect the experimentally observed formation of Cu_2_O.^96,97^ Similarly, the oxidized Cu(110) also consists of the parallel, added-row Cu–O chains, whose simulated images are consistent with both experimental STM and TEM observation as shown in Fig. 5(j–n).^81,82,98^

## Conclusions

In summary, we present a new strategy for global structural optimization using a HEA that combines DE and GA with a “tribe” framework. This algorithm combines the ability of the GA to explore the PES and the ability of the DE to exploit the PES. In practice, the HEA program performs better than well-established methods for optimizing surface systems. The high efficiency stems from the newly introduced DE operators that are effective in generating lower energy configurations, an efficient GP regressor that expedites the identification of low-lying structures, and a multi-tribe framework that maintains a high structural diversity. We demonstrate the efficacy of the HEA method in obtaining the complex surface oxide structure of different facets of Pt, Pd, and Cu. The optimized structures are lower than previously reported models and are consistent with experimental observation. The newly proposed HEA program may open a new avenue for the study of the complex reconstruction of heterogeneous catalysts under reaction conditions.

## Data availability

The data that support the findings of this study are available within the article and its ESI,† or from the corresponding author on reasonable request.

## Author contributions

Xiangcheng Shi: methodology; investigation; visualization; formal analysis; validation; writing – original draft. Dongfang Cheng: methodology; investigation; formal analysis; writing – original draft. Ran Zhao: methodology; formal analysis. Gong Zhang: investigation; formal analysis. Shican Wu: methodology; formal analysis; Shiyu Zhen: visualization; formal analysis; Zhi-Jian Zhao: supervision; writing – review & editing; resources and funding acquisition. Jinlong Gong: supervision; formal analysis; writing – review & editing; resources and funding acquisition.

## Conflicts of interest

There are no conflicts to declare.

## Supplementary Material

SC-014-D3SC02974C-s001

## References

[cit1] Zhang J., Xu Q., Wang J., Hu Y., Jiang H., Li C. (2022). Sci. China Mater..

[cit2] Lin X.-M., Yang X.-T., Chen H.-N., Deng Y.-L., Chen W.-H., Dong J.-C., Wei Y.-M., Li J.-F. (2023). J. Energy Chem..

[cit3] Ge R., Li J., Duan H. (2022). Sci. China Mater..

[cit4] Liu B., Liu G., Tang Y., Cheng H.-M. (2022). Sci. China Mater..

[cit5] Qian K., Yu Z., Liu Y., Gosztola D. J., Winans R. E., Cheng L., Li T. (2022). J. Energy Chem..

[cit6] Zugic B., Wang L., Heine C., Zakharov D. N., Lechner B. A. J., Stach E. A., Biener J., Salmeron M., Madix R. J., Friend C. M. (2016). Nat. Mater..

[cit7] Ma Z., Li J., Ling T. (2022). Trans. Tianjin Univ..

[cit8] Guo W., Luo H., Fang D., Jiang Z., Chi J., Shangguan W. (2022). J. Energy Chem..

[cit9] Cai J., Yang Z., Zhou X., Wang B., Suzana A., Bai J., Liao C., Liu Y., Chen Y., Song S., Zhang X., Wang L., He X., Meng X., Karami N., Ali Shaik Sulaiman B., Chernova N. A., Upreti S., Prevel B., Wang F., Chen Z. (2023). J. Energy Chem..

[cit10] Behrens M., Studt F., Kasatkin I., Kühl S., Hävecker M., Abild-Pedersen F., Zander S., Girgsdies F., Kurr P., Kniep B.-L., Tovar M., Fischer R. W., Nørskov J. K., Schlögl R. (2012). Science.

[cit11] Tao F., Grass M. E., Zhang Y., Butcher D. R., Renzas J. R., Liu Z., Chung J. Y., Mun B. S., Salmeron M., Somorjai G. A. (2008). Science.

[cit12] Liu S., Yang C., Zha S., Sharapa D., Studt F., Zhao Z. J., Gong J. (2021). Angew. Chem., Int. Ed..

[cit13] Tan Z., Li Y., Xi X., Jiang S., Li X., Shen X., Zhang P., He Z. (2023). Nano Res..

[cit14] Gan Q., Qin N., Li Z., Gu S., Liao K., Zhang K., Lu L., Xu Z., Lu Z. (2022). Nano Res..

[cit15] Grimaud A., Demortière A., Saubanère M., Dachraoui W., Duchamp M., Doublet M.-L., Tarascon J.-M. (2016). Nat. Energy.

[cit16] Zheng Y.-R., Vernieres J., Wang Z., Zhang K., Hochfilzer D., Krempl K., Liao T.-W., Presel F., Altantzis T., Fatermans J., Scott S. B., Secher N. M., Moon C., Liu P., Bals S., Van Aert S., Cao A., Anand M., Nørskov J. K., Kibsgaard J., Chorkendorff I. (2021). Nat. Energy.

[cit17] Lee A. F., Ellis C. V., Naughton J. N., Newton M. A., Parlett C. M. A., Wilson K. (2011). J. Am. Chem. Soc..

[cit18] Polo-Garzon F., Bao Z., Zhang X., Huang W., Wu Z. (2019). ACS Catal..

[cit19] Fuchs T., Drnec J., Calle-Vallejo F., Stubb N., Sandbeck D. J. S., Ruge M., Cherevko S., Harrington D. A., Magnussen O. M. (2020). Nat. Catal..

[cit20] Miller D. J., Öberg H., Kaya S., Sanchez Casalongue H., Friebel D., Anniyev T., Ogasawara H., Bluhm H., Pettersson L. G. M., Nilsson A. (2011). Phys. Rev. Lett..

[cit21] Hao J., Xie S., Huang Q., Ding Z., Sheng H., Zhang C., Yao J. (2022). CCS Chem..

[cit22] Zhang J., Glezakou V. A. (2020). Int. J. Quantum Chem..

[cit23] Jäger M., Schäfer R., Johnston R. L. (2018). Adv. Phys.: X.

[cit24] Khatun M., Majumdar R. S., Anoop A. (2019). Front. Chem..

[cit25] Grajciar L., Heard C. J., Bondarenko A. A., Polynski M. V., Meeprasert J., Pidko E. A., Nachtigall P. (2018). Chem. Soc. Rev..

[cit26] Musa E., Doherty F., Goldsmith B. R. (2022). Curr. Opin. Chem. Eng..

[cit27] Sierka M. (2010). Prog. Surf. Sci..

[cit28] Merte L. R., Bisbo M. K., Sokolović I., Setvín M., Hagman B., Shipilin M., Schmid M., Diebold U., Lundgren E., Hammer B. (2022). Angew. Chem., Int. Ed..

[cit29] Deaven D. M., Ho K. M. (1995). Phys. Rev. Lett..

[cit30] Lyakhov A. O., Oganov A. R., Valle M. (2010). Comput. Phys. Commun..

[cit31] Gruznev D. V., Bondarenko L. V., Matetskiy A. V., Tupchaya A. Y., Chukurov E. N., Hsing C. R., Wei C. M., Eremeev S. V., Zotov A. V., Saranin A. A. (2015). Phys. Rev. B: Condens. Matter Mater. Phys..

[cit32] Chou J. P., Wei C. M., Wang Y. L., Gruznev D. V., Bondarenko L. V., Matetskiy A. V., Tupchaya A. Y., Zotov A. V., Saranin A. A. (2014). Phys. Rev. B: Condens. Matter Mater. Phys..

[cit33] Gruznev D. V., Bondarenko L. V., Tupchaya A. Y., Yakovlev A. A., Mihalyuk A. N., Zotov A. V., Saranin A. A. (2018). Surf. Sci..

[cit34] Gruznev D. V., Eremeev S. V., Bondarenko L. V., Tupchaya A. Y., Yakovlev A. A., Mihalyuk A. N., Chou J.-P., Zotov A. V., Saranin A. A. (2018). Nano Lett..

[cit35] Wang Q., Oganov A. R., Zhu Q., Zhou X.-F. (2014). Phys. Rev. Lett..

[cit36] Zakaryan H. A., Kvashnin A. G., Oganov A. R. (2017). Sci. Rep..

[cit37] Kvashnin A. G., Kvashnin D. G., Oganov A. R. (2019). Sci. Rep..

[cit38] Revard B. C. , Tipton W. W. and Hennig R. G., in Prediction and Calculation of Crystal Structures, 2014, ch. 489, pp. 181–222, 10.1007/128_2013_489 24515753

[cit39] Shi X., Lin X., Luo R., Wu S., Li L., Zhao Z.-J., Gong J. (2021). JACS Au.

[cit40] Lyakhov A. O., Oganov A. R., Stokes H. T., Zhu Q. (2013). Comput. Phys. Commun..

[cit41] Jørgensen M. S., Larsen U. F., Jacobsen K. W., Hammer B. (2018). J. Phys. Chem. A.

[cit42] Cheng G., Gong X.-G., Yin W.-J. (2022). Nat. Commun..

[cit43] Zuo Y., Qin M., Chen C., Ye W., Li X., Luo J., Ong S. P. (2021). Mater. Today.

[cit44] Palizhati A., Zhong W., Tran K., Back S., Ulissi Z. W. (2019). J. Chem. Inf. Model..

[cit45] Yao M., Ji J., Li X., Zhu Z., Ge J.-Y., Singh D. J., Xi J., Yang J., Zhang W. (2023). Sci. China Mater..

[cit46] Li H., Jiao Y., Davey K., Qiao S.-Z. (2023). Angew. Chem., Int. Ed..

[cit47] Kaappa S., del Río E. G., Jacobsen K. W. (2021). Phys. Rev. B.

[cit48] Hajinazar S., Sandoval E. D., Cullo A. J., Kolmogorov A. N. (2019). Phys. Chem. Chem. Phys..

[cit49] Pickard C. J., Needs R. J. (2011). J. Phys.: Condens. Matter.

[cit50] Huo H., Rupp M. (2022). Mach. learn.: sci. technol..

[cit51] Vilhelmsen L. B., Hammer B. (2014). J. Chem. Phys..

[cit52] Fan T.-E., Shao G.-F., Ji Q.-S., Zheng J.-W., Liu T.-d., Wen Y.-H. (2016). Comput. Phys. Commun..

[cit53] Chanussot L., Das A., Goyal S., Lavril T., Shuaibi M., Riviere M., Tran K., Heras-Domingo J., Ho C., Hu W., Palizhati A., Sriram A., Wood B., Yoon J., Parikh D., Zitnick C. L., Ulissi Z. (2021). ACS Catal..

[cit54] Sriram A. , Das A., Wood B. M., Goyal S. and Zitnick C. L., arXiv, 2022, preprint, arXiv:2203.09697, 10.48550/arXiv.2203.09697

[cit55] Gardner J. R. , Pleiss G., Bindel D., Weinberger K. Q. and Wilson A. G., arXiv, 2018, preprint, arXiv:1809.11165, 10.48550/arXiv.1809.11165

[cit56] Himanen L., Jäger M. O. J., Morooka E. V., Federici Canova F., Ranawat Y. S., Gao D. Z., Rinke P., Foster A. S. (2020). Comput. Phys. Commun..

[cit57] Rasmussen C. E. and Williams C. K. I., Gaussian processes for machine learning, MIT Press, Cambridge, Mass, 2006

[cit58] Simm G. N., Reiher M. (2018). J. Chem. Theory Comput..

[cit59] Bisbo M. K., Hammer B. (2020). Phys. Rev. Lett..

[cit60] Moriconi R., Kumar K. S. S., Deisenroth M. P. (2019). Optim. Lett..

[cit61] Manzhos S., Ihara M. (2023). J. Chem. Phys..

[cit62] Shahriari B., Swersky K., Wang Z., Adams R. P., de Freitas N. (2016). Proc. IEEE.

[cit63] Hernandez-Lobato J. M., Requeima J., Pyzer-Knapp E. O., Aspuru-Guzik A. (2017). Proc. Mach. Learn. Res..

[cit64] Arrigoni M., Madsen G. K. H. (2021). Npj Comput. Mater..

[cit65] Huang S.-D., Shang C., Zhang X.-J., Liu Z.-P. (2017). Chem. Sci..

[cit66] Deringer V. L., Bartók A. P., Bernstein N., Wilkins D. M., Ceriotti M., Csányi G. (2021). Chem. Rev..

[cit67] Banerjee A., Dunson D. B., Tokdar S. T. (2012). Biometrika.

[cit68] Grajciar L., Heard C. J., Bondarenko A. A., Polynski M. V., Meeprasert J., Pidko E. A., Nachtigall P. (2018). Chem. Soc. Rev..

[cit69] Zhang P., Wang T., Gong J. (2023). CCS Chem..

[cit70] Liu S., Zong J., Zhao Z.-J., Gong J. (2020). GreenChE.

[cit71] Li L., Liu T., Zhou Z., Guo P., Li X., Wu S. (2022). Sci. China Mater..

[cit72] Kang H., Zhang Y., Wu Y., Hu S., Li J., Chen Z., Sui Y., Wang S., Zhao S., Xiao R., Yu G., Peng S., Jin Z., Liu X. (2022). Sci. China Mater..

[cit73] Duan Z., Henkelman G. (2021). ACS Catal..

[cit74] Hawkins J. M., Weaver J. F., Asthagiri A. (2009). Phys. Rev. B: Condens. Matter Mater. Phys..

[cit75] Devarajan S. P., Hinojosa J. A., Weaver J. F. (2008). Surf. Sci..

[cit76] Holby E. F., Greeley J., Morgan D. (2012). J. Phys. Chem. C.

[cit77] van Spronsen M. A., Frenken J. W. M., Groot I. M. N. (2017). Nat. Commun..

[cit78] Friebel D., Miller D. J., O'Grady C. P., Anniyev T., Bargar J., Bergmann U., Ogasawara H., Wikfeldt K. T., Pettersson L. G. M., Nilsson A. (2011). Phys. Chem. Chem. Phys..

[cit79] Zheng G., Altman E. I. (2000). Surf. Sci..

[cit80] Mönig H., Todorović M., Baykara M. Z., Schwendemann T. C., Rodrigo L., Altman E. I., Pérez R., Schwarz U. D. (2013). ACS Nano.

[cit81] Liu Q., Li L., Cai N., Saidi W. A., Zhou G. (2014). Surf. Sci..

[cit82] Li M., Curnan M. T., Saidi W. A., Yang J. C. (2022). Nano Lett..

[cit83] Over H., Seitsonen A. P. (2002). Science.

[cit84] Jiang G., Han D., Han Z., Gao J., Wang X., Weng Z., Yang Q.-H. (2022). Transactions of Tianjin University.

[cit85] Deng B., Zhao X., Li Y., Huang M., Zhang S., Dong F. (2022). Sci. China Chem..

[cit86] Zemlyanov D., Aszalos-Kiss B., Kleimenov E., Teschner D., Zafeiratos S., Hävecker M., Knop-Gericke A., Schlögl R., Gabasch H., Unterberger W., Hayek K., Klötzer B. (2006). Surf. Sci..

[cit87] Jin T., Chen F., Guo L., Tang Q., Wang J., Pan B., Wu Y., Yu S. (2021). J. Phys. Chem. C.

[cit88] Weissman-Wenocur D. L., Shek M. L., Stefan P. M., Lindau I., Spicer W. E. (1983). Surf. Sci..

[cit89] Li L., Mi X., Shi Y., Zhou G. (2012). Phys. Rev. Lett..

[cit90] Kunze S., Tănase L. C., Prieto M. J., Grosse P., Scholten F., de Souza Caldas L., van Vörden D., Schmidt T., Cuenya B. R. (2021). Chem. Sci..

[cit91] Kangas T., Laasonen K. (2012). Surf. Sci..

[cit92] Lahtonen K., Hirsimäki M., Lampimäki M., Valden M. (2008). J. Chem. Phys..

[cit93] Lampimäki M., Lahtonen K., Hirsimäki M., Valden M. (2007). J. Chem. Phys..

[cit94] Saidi W. A., Lee M., Li L., Zhou G., McGaughey A. J. H. (2012). Phys. Rev. B: Condens. Matter Mater. Phys..

[cit95] Tjung S. J., Zhang Q., Repicky J. J., Yuk S. F., Nie X., Santagata N. M., Asthagiri A., Gupta J. A. (2019). Surf. Sci..

[cit96] Lee M., McGaughey A. J. H. (2011). Phys. Rev. B: Condens. Matter Mater. Phys..

[cit97] Cao J., Rinaldi A., Plodinec M., Huang X., Willinger E., Hammud A., Hieke S., Beeg S., Gregoratti L., Colbea C., Schlögl R., Antonietti M., Greiner M., Willinger M. (2020). Nat. Commun..

[cit98] Li Y., Chen H., Wang W., Huang W., Ning Y., Liu Q., Cui Y., Han Y., Liu Z., Yang F., Bao X. (2020). Nano Res..

