# Supplementary material for: Accessing complex reconstructed material structures with hybrid global optimization accelerated via on-the-fly machine learning
Source: Chem Sci. 2023 Jul 20;14(33):8777–84. doi: 10.1039/d3sc02974c (PMC10445438; doi:10.1039/d3sc02974c)
Supplement: SC-014-D3SC02974C-s001 [file SC-014-D3SC02974C-s001.pdf]

Electronic Supplementary Material (ESI) for Chemical Science.
This journal is © The Royal Society of Chemistry 2023

Supplemental Materials

Accessing Complex Reconstructed Material Structures with Hybrid Global Optimization Accelerated via on-the-fly Machine Learning

Xiangcheng Shi, Dongfang Cheng, Ran Zhao, Gong Zhang, Shican Wu, Shiyu Zhen, Zhi-Jian Zhao* and Jinlong Gong*

1. Computational Methods

1.1 DFT calculations

Density functional theory (DFT) calculations were performed with the plane-wave-based Vienna Ab Initio Simulation package, VASP[1]. The electron exchange and correlation were described by the generalized gradient approximation (GGA) in the form of the Perdew-Burke-Ernzerhof (PBE) functional[2]. Electron-ion interactions were described within the projector-augmented wave framework[3]. In all calculations, the energy cutoff of the plane-wave basis set was 400 eV. Optimized structures were obtained by minimizing the forces on each ion until they fell below 0.02 eV/Å.

We employed the HEA approach implemented to perform global optimization of structures of metal surface oxides. During the optimization, slab models consisting of four metal layers using a (4×4) supercell were employed to model the metal surface (the bottom two layers were fixed), with the Brillouin zone being sampled by the Gamma point only. A 20 Å vacuum gap was added to eliminate interactions between periodic images perpendicular to the slab surface. The obtained low-lying structures were further checked and calculated with the Brillouin zone integration in k-space being performed on a 4×4×1 k-point mesh sampled using the Monkhorst-Pack scheme[4]. A Bader charge analysis of surface metal atoms was then carried out for the oxidation-state analysis by calculating the Bader charges of bulk metal oxides as references [5]. The STM is generated using the partial charge densities and p4vasp software.

1.2 The formalism of formation energy

The formalism of formation energy (E_θ) was adopted using the following reaction[6, 7]:

$$M_{slab} + nH_2O \rightarrow MO(\theta) + 2nH^+ + 2ne^-$$

n is the number of oxygens that are adsorbed to the pure metal slab and fractional coverage, θ, is given by $\theta = \frac{n}{n_{Pt}}$. n_{Pt} is the number of metal surface atoms in the metal slab. Following this formalism, the formation energy (unit: eV/O-site) of oxidized metal surface with different coverage was calculated by:

$$E_\theta = \frac{\left(E_{MO(\theta)} + nE_{H_2}\right) - \left(E_{M_{slab}} + nE_{H_2O}\right)}{n}$$

For comparing structures with different number of metal and O atom, the following equation was used to calculate the formation energy (unit: eV/atoms):

$$E_\theta = \frac{E(M_mO_n) - m * E(M) - n * E(O)}{m + n}$$

where $E(M)$ and $E(O)$ represents the energy of isolated metal and oxygen atom, respectively.

1.3 The quantified evolution of offspring operator

To study the efficiency of the offspring operator used in the GA search, how the different offspring operators affect the energy of the offspring structure for the 0.75 ML O-Pt(111) surface is studied. Noted that mutations may be applied after a new candidate is generated through the GA operator. Following Behler *et al.*, the energy of a offspring structure is expressed relative to the parents' average energy, as given by[8]

$$P = 100 * (\frac{E_{candidate}}{\bar{E}_{parent}} - 1)$$

where $E_{candidate}$ is the formation energy of the offspring candidate and $\bar{E}_{parent}$ is the average formation energy of its parents. If the offspring candidate has lower energy than the average of its parents, P is negative, and we can say that the offspring operator was "successful" in progressing the structural search.

1.4 Kendall's τ coefficient

For a Gaussian process-based sampling, apart from the model accuracy, a relatively correct ranking of the excessive offspring candidates is also desired. The reliable sampling can be quantified using Kendall's τ coefficient.

The definition of Kendall's τ coefficient of two arrays *x* and *y* that is used is:

$$\tau = \frac{P - Q}{\sqrt{(P + Q + T) * (P + Q + U)}}$$

where P is the number of concordant pairs, Q the number of discordant pairs, T the number of ties only in *x*, and U the number of ties only in *y*. If a tie occurs for the same pair in both *x* and *y*, it is not added to either T or U.

2. Supplemental Figures and Tables

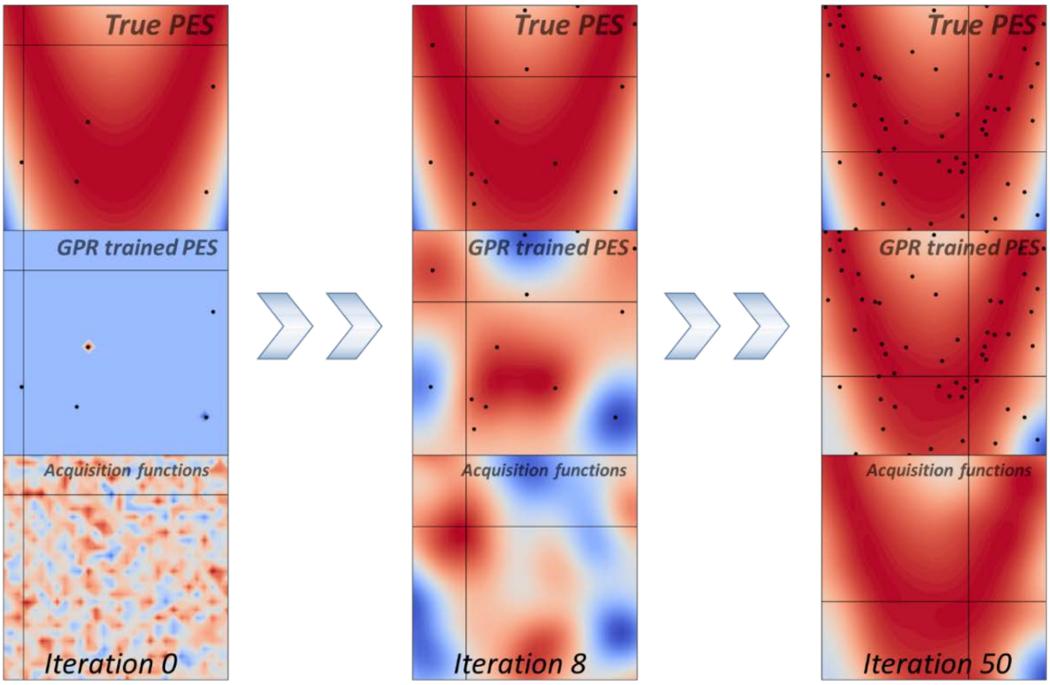

Fig. S1 Example of a surrogate guided structure searches in a non-convex search space. To simulate the non-convexity of PES, the Rosenbrock function is used as an illustrative example. The true PES is shown in the top row. The GPR on-the-fly trained PES is shown in the middle row, with the training samples shown in the black dot. The result of TS acquisition functions is shown in the bottom row. Red colors signify regions with low values while blue colors signify regions with correspondingly high values.

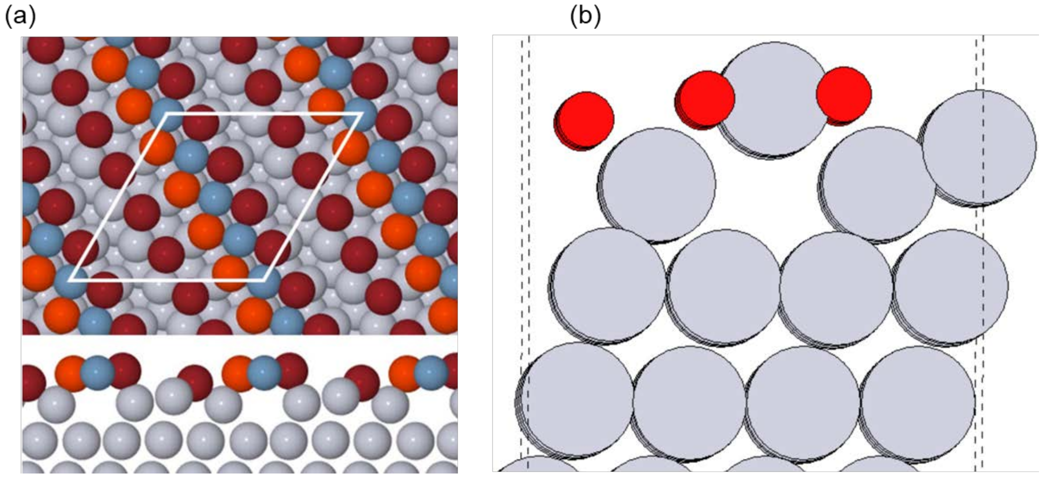

Fig. S2 (a) Top and side views of the most stable surface O atom configurations at 0.75 ML O-Pt(111) proposed by Hawkins *et al.* [9] (Reproduced with permission from Refs [9]. Copyright 2009 American Physical Society). (b) The corresponding model of Hawkins *et al.*'s structure. Noted that this structure is also found during our global structural optimization, not manually built.

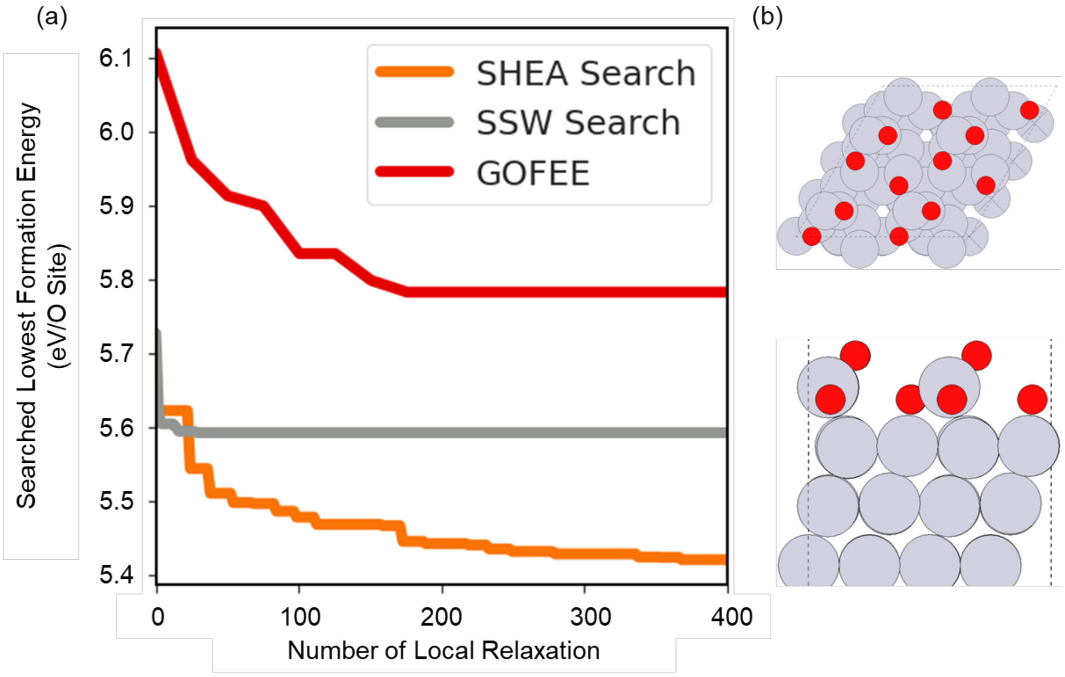

Fig. S3 (a) The energy learning curves of (2×2) 0.75 ML O Pt(111) surface. All searches drawn here are both repeated the search three times of which the plotted line represents the mean value. (b) The globally optimized (2×2) 0.75 ML O Pt(111) surface after repeated to 4×4 supercell. The stability of this structure is 0.28 eV/O-site higher in formation energy (3.43 eV higher in potential energy) than that directly obtained by optimizing the (4×4) surface.

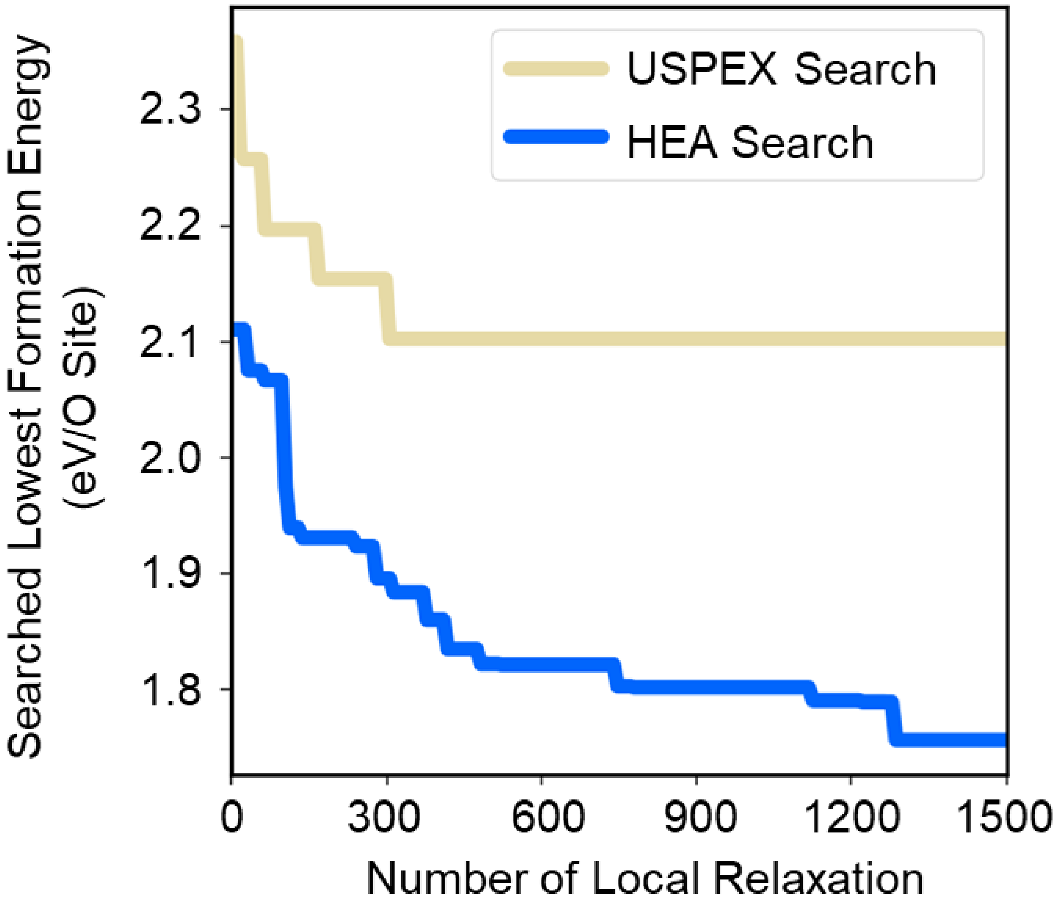

Fig. S4 Structural optimizing performance of USPEX and HEA program on (4×4) 1.0 ML O-Pd(111).

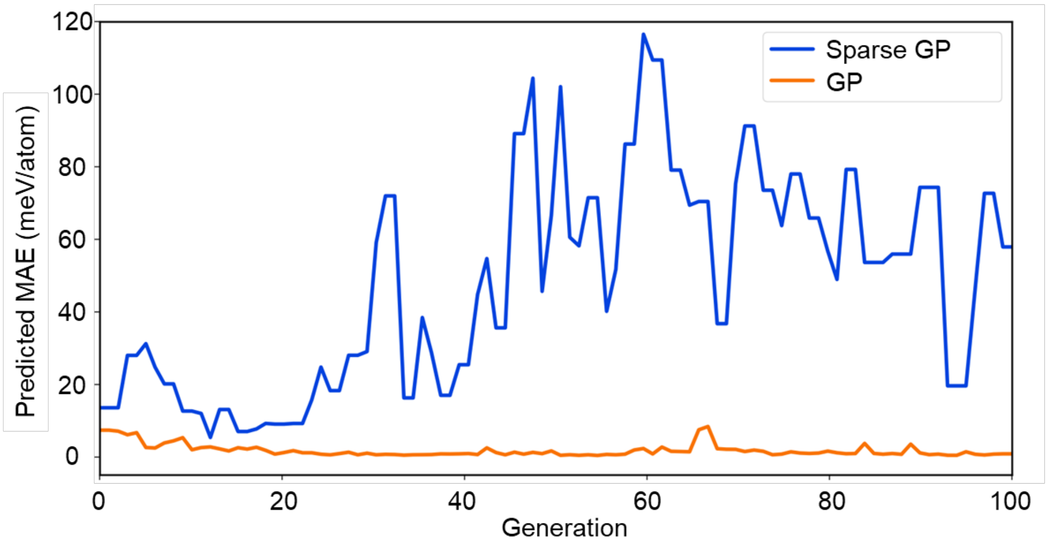

Fig. S5 The on-the-fly predict accuracy in optimizing O-Pt(111) using GP Regressor with full covariance matrix and sparse GP Regressor. Here both regressors are trained using relaxed structures with their relaxed energy separately inside each tribe.

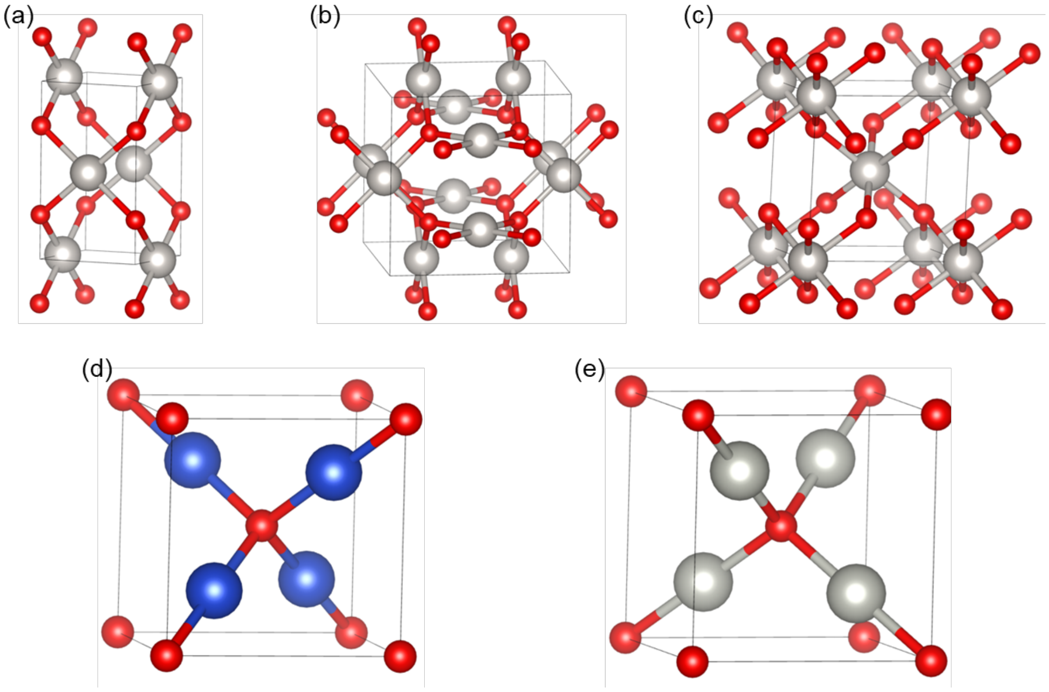

Fig. S6 Structures of bulk metal oxides: (a) PtO; (b) Pt_3O_4; (c) PtO_2; (d) Cu_2O; (e) Pd_2O.

Reference:

1. J. P. Perdew, W. Yang, K. Burke, Z. Yang, E. K. U. Gross, M. Scheffler, G. E. Scuseria, T. M. Henderson, I. Y. Zhang, A. Ruzsinszky, H. Peng, J. Sun, E. Trushin and A. Görling, *Proceedings of the National Academy of Sciences*, 2017, **114**, 2801-2806.
2. J. P. Perdew, K. Burke and M. Ernzerhof, *Physical Review Letters,* 1996, **77**, 3865-3868.
3. P. E. Blöchl, *Physical Review B*, 1994, **50**, 17953-17979.
4. H. J. Monkhorst and J. D. Pack, *Physical Review B,* 1976, **13**, 5188-5192.
5. Z. Duan and G. Henkelman, *ACS Catalysis*, 2021, **11**, 14439-14447.
6. J. K. Nørskov, J. Rossmeisl, A. Logadottir, L. Lindqvist, J. R. Kitchin, T. Bligaard and H. Jónsson, *The Journal of Physical Chemistry B*, 2004, **108**, 17886-17892.
7. E. F. Holby, J. Greeley and D. Morgan, *The Journal of Physical Chemistry C*, 2012, **116**, 9942-9946.
8. M. L. Paleico and J. Behler, *The Journal of Chemical Physics*, 2020, **153**, 054704.
9. J. M. Hawkins, J. F. Weaver and A. Asthagiri, *Physical Review B*, 2009, **79**, 125434.
